# Association of Calf Circumference with Clinical and Biochemical Markers in Older Adults with COVID-19 Admitted at Intensive Care Unit: A Retrospective Cross-Sectional Study

**DOI:** 10.3390/diseases12050097

**Published:** 2024-05-08

**Authors:** Vanessa A. Araújo, Jefferson S. Souza, Bruna M. Giglio, Patrícia C. B. Lobo, Gustavo D. Pimentel

**Affiliations:** Faculty of Nutrition, Federal University of Goiás, Goiânia 74605080, Brazil; vanenut95@gmail.com (V.A.A.); jefferson.nutricao@hotmail.com (J.S.S.); brunamgiglio@gmail.com (B.M.G.); patriciacristina.nutri@gmail.com (P.C.B.L.)

**Keywords:** anthropometry, calf circumference, COVID-19, older adults, intensive care unit

## Abstract

Background: COVID-19 is an infectious disease characterized by a severe catabolic and inflammatory state, leading to loss of muscle mass. The assessment of muscle mass can be useful to identify nutritional risk and assist in early management, especially in older adults who have high nutritional risks. The aim of this study was to evaluate the association of calf circumference (CC) with clinical and biochemical markers and mortality in older adults with COVID-19 admitted to the intensive care unit (ICU). Methods: A retrospective cross-sectional study was conducted in a public hospital. CC was adjusted for body mass index (BMI), reducing 3, 7, or 12 cm for a BMI of 25–29.9, 30–39.9, and ≥40 kg/m^2^, respectively, and classified as reduced when <33 cm for women and <34 cm for men. Pearson’s correlation between BMI and CC was performed to assess the association between variables. Regression analysis was adjusted for sex, age, and BMI variables. Cox regression was used to assess survival related to CC. Results: A total of 208 older adults diagnosed with COVID-19 admitted to ICU were included, of which 84% (*n* = 176) were classified as having reduced CC. These patients were older, with lower BMI, higher nutritional risk, malnourished, and higher concentration of urea and urea–creatinine ratio (UCR) compared with the group with normal CC. There was an association between edematous patients at nutritional risk and malnourished with reduced CC in the Cox regression, either adjusted or not for confounding. Conclusions: CC was not associated with severity, biochemical markers, or mortality in older adults with COVID-19 admitted to the ICU, but it was associated with moderately malnourished patients assessed by subjective global assessment (SGA).

## 1. Introduction

Older people infected with severe acute respiratory coronavirus 2 (SARS-CoV-2) are more susceptible and have a high incidence of severe disease and mortality, requiring ventilatory support and hospitalization in intensive care units (ICUs) [1,2]. SARS-CoV-2 induces severe inflammatory stress [3], as the effects caused by the virus and viral evasion on the host’s immune response play an important role in disease severity [4]. The acute inflammatory response to infection leads to an increase in inflammatory markers, such as hematological markers, white blood cell count, C-reactive protein (CRP), and the neutrophil–lymphocyte ratio (NLR), which has been shown to be a biomarker of systemic inflammation and is positively associated with coronavirus disease 2019 (COVID-19) [5].

This hypermetabolic response leads to increased energy expenditure and the release of energy substrate from muscle protein stores, leading to loss of muscle mass, excessive generation of urea, and reduced excretion of creatinine, impairing the immune response [2]. Skeletal muscle serves as a source of amino acids for maintaining protein synthesis and preserving vital tissues and organs during stress conditions [6]. Additionally, elevated creatinine urea nitrogen (UCR) accompanies the loss of skeletal muscle mass and may be a potential indicator of ongoing muscle catabolism in critically ill patients [7]; there are no studies evaluating UCR in patients with COVID-19.

The degradation of muscle mass in patients with COVID-19 may be greater in older people and patients admitted to the ICU, because of bed rest, use of antivirals, systemic inflammation, and length of stay [8,9,10]. The assessment of muscle mass can be useful in the hospital routine to guide adjustments in nutritional therapy and clinical prognosis during ICU stay [2], being recommended in the evaluation of patients with COVID-19 [11,12]. There are several techniques that are validated and can be used as a muscle mass marker, such as DXA, computed tomography, and magnetic resonance [9,13]. However, they are not always practical, require technical support, and are expensive [2,13]. A simple and practical method that can be used as a marker of muscle mass in older people is calf circumference (CC) [2], widely used in clinical practice due to its feasibility and ease of execution [11] and recommended by the European consensus [14]. Some studies have been carried out to assess muscle mass in patients with COVID-19 using computed tomography [2,15,16]. There are no studies evaluating CC with inflammatory, clinical, or mortality parameters in older patients with COVID-19.

Thus, we hypothesize that reduced CC in older patients with COVID-19 may be associated with high inflammation and increased clinical parameters of severity and serve as a nutritional assessment used in the ICU routine for mortality, as it is a simple, economical, and easy-to-apply method in hospital units [17]. Therefore, this article aims to evaluate CC as a marker for monitoring inflammation, clinical parameters, and mortality in older patients diagnosed with COVID-19 admitted to the ICU. Our study will be the first to evaluate the association of CC with inflammatory and clinical parameters of severity and nutritional assessment.

## 2. Methods

### 2.1. Study and Sample

A retrospective cross-sectional study was carried out at the Clinical Hospital of the Federal University of Goiás, a field hospital for patients diagnosed with COVID-19 in the city of Goiânia—Brazil. Data collection was carried out by reviewing electronic medical records of patients diagnosed with COVID-19 between October 2020 and September 2021. This study was submitted to and approved by the Ethics and Research Committee of the Clinical Hospital of UFG under protocol numbers: 5.053.025 and 5.120.133. As it is a retrospective study, the waiving of the term free and informed consent was requested and accepted, as this study used secondary data obtained from material already collected and authorized from electronic medical records of patients hospitalized in the intensive care unit.

Patients admitted to the ICU diagnosed with COVID-19 confirmed by real-time reverse transcriptase polymerase chain reaction (RT-PCR) and CT scan, gold standard methods for detecting COVID-19 [18], with a minimum hospital stay of 24 h and who were or were not using mechanical ventilation, vasoactive drugs, or sedation, were considered eligible for this research, and a review of medical records was performed. Patients were aged ≥ 60 years. Those who were in palliative care, using a dose greater than 2 mcg/kg/day of vasoactive drugs, and did not have biochemical tests or anthropometric data in their medical records were excluded.

### 2.2. Data Collection

Data collection was performed by reviewing electronic medical records. Data, such as demographic characteristics such as age and gender of the patient, were collected from the patient’s day of admission to the ICU. Clinical data, such as days of hospitalization in the ICU, independent of the duration of prior hospitalization, comorbidities, use of mechanical ventilation, use of vasoactive drugs, sedation, hemodialysis, or lower limb edema, were graded on a scale of 1+ to 4+ based on the depth of the indentation and the time taken to return to baseline [19] and mortality. Disease severity scores were also collected, such as Sequential Organ Failure Assessment (SOFA score) [20], Acute Physiology and Chronic Health Inquiry (APACHE II score) [21], and nutritional status variables, such as Nutrition Risk in Critically Ill (NUTRIC) [11,22], Subjective Global Assessment (SGA) [23], and Nutritional Risk Screening (NRS-2002) [11,24]. All data collection was carried out simultaneously upon admission.

Biochemical analysis took place according to the hospital’s routine. Data such as absolute count of hematocrit (HT), hemoglobin (HB), neutrophils and lymphocytes to obtain the NLR, urea and creatinine to obtain the UCR, CRP, and lactate were extracted from the biochemical tests. Regarding anthropometric data, data such as height (m), weight (kg), CC (cm), and BMI (kg/m^2^) were collected from the medical records. The ratio between body weight and height squared was used to calculate BMI [25]. BMI was classified as underweight (≤22 kg/m^2^), adequate or eutrophic (<22 and >27 kg/m^2^), and overweight (≥27 kg/m^2^) [26]. CC was assessed on the day of ICU admission and was adjusted for BMI, reducing by 3, 7, or 12 cm for a BMI of 25–29.9, 30–39.9, and ≥40 kg/m^2^, respectively [24]; we also performed CC analysis without BMI adjustment (Supplementary Data). CC was classified as reduced when <33 for women and <34 for men [17]. The anthropometric measurements performed upon admission to the ICU at the Clinical Hospital of UFG are conducted daily by trained professionals. We classified patients with an NRS score ≥3 [25] as at nutritional risk and SGA patients as well-nourished, moderately malnourished, and severely malnourished [23].

### 2.3. Statistical Analyzes

There was no sample calculation because the patients were collected for convenience through the medical records. The Shapiro–Wilk test was used to verify data normality. Variables were presented as median and interquartile range, value, and percentage. Differences between groups were applied using the Mann–Whitney U test or Chi-square. Pearson’s correlation was performed to assess the association between BMI and CC. The automated binary regression test was performed considering adjustments for sex, age, and BMI to assess the association between variables. The variables were described considering the odds ratio and the confidence interval. Finally, the association between CC and mortality was examined using the Cox regression model. SPSS software (version 20.0) was used for all analyses, and values were considered significant when *p* < 0.05. Pearson’s correlation graph was performed using the R software (version 4.2.2).

## 3. Results

A total of 591 patients were eligible for the study, of which 356 were excluded because they were not admitted to the ICU and 27 because they did not have CC values, totaling 208 patients. Among the 208 (113M/95F) older adults admitted to the ICU with COVID-19, the mean age was 72 years, 59% (*n* = 124) of the sample were hypertensive, with a mean of 10 days of hospitalization, 43% (*n* = 90) of the sample were on mechanical ventilation, 25% (*n* = 52) were using vasoactive drugs, 30% (*n* = 63) were using sedatives, 97% (*n* = 203) were at nutritional risk, 49% (*n* = 103) were malnourished, 84% (*n* = 176) were classified as having reduced CC, and 65% (*n* = 137) died (Table 1).

Although there was no significant difference in HT, HB, creatinine, neutrophil, lymphocyte, NLR, CRP, lactate, SOFA score, APACHE II score, NUTRIC, continuous NRS, invasive mechanical ventilation, vasoactive drugs, sedation, hemodialysis, death and comorbidities such as diabetes, chronic obstructive pulmonary disease, chronic kidney disease, cardiomyopathy, dementia, dyslipidemia, or cancer between groups (Table 1), patients in the low CC group (women CC < 33 cm and men CC < 34 cm) were older, predominantly male, had fewer days of hospitalization in the ICU, lower BMI, higher urea concentration and urea–creatinine ratio, and higher nutritional risk and quantity of comorbidities and malnourishment than the normal CC group (whether in women with >33 cm or in men >34 cm CC) (Table 1).

When performing the stratification of the patients by sex and CC (reduced and normal), we observed that for females in the CC <33 cm group, patients were older, with higher nutritional risk and more undernourishment, as well as lower weight and BMI and more hypertension than the group with CC >33 cm. For males, we found that the CC <34 cm group was also older, with higher nutritional risk, more undernourishment, shorter lengths of stay, lower weight and BMI, and higher UCR than the CC >34 cm group (Table 2).

**Figure 1:**
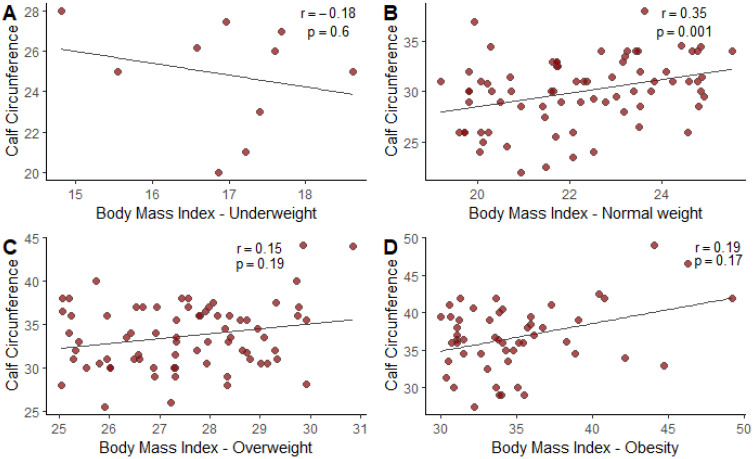
(**A**): Pearson correlation graph between BMI and CC of patients admitted to the ICU with COVID-19. (**B**): Pearson correlation chart between underweight BMI and CC of patients admitted to the ICU with COVID-19. (**C**): Pearson correlation chart between normal BMI and CC of patients admitted to the ICU with COVID-19. (**D**): Pearson correlation chart between BMI overweight/obesity and CC of patients admitted to the ICU with COVID-19. p = *p*-value, r = pearson correlation coefficient.

In addition, we observed a weak correlation between BMI with underweight and CC (r= −0.18; *p*= 0.6), BMI and CC with overweight (r = 0.15; *p*= 0.19), and BMI with obesity (r = 0.19; *p* = 0.17), and a moderate correlation between normal BMI and CC (r = 0.35; *p* = 0.001) (Figure 1).

**Figure 2:**
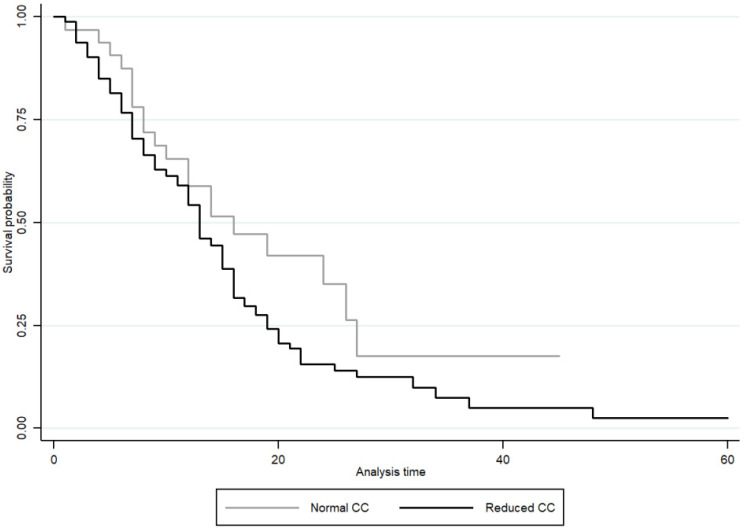
Survival curve of patients admitted to ICU with COVID-19 by calf circumference classification. Crude Cox model.

In the association analysis, there was a greater chance of CC reduction the greater the edema (OR 0.34; CI 0.13–0.89) and nutritional risk (OR 1.49; CI 1.06–2.10). After adjusting for confounding variables, these associations were maintained (Table 3). We also observed that malnourished individuals are more likely to have a reduced CC than healthy individuals according to the SGA assessment (OR 2.32; CI 1.02–5.25); even after adjusting for confounding variables, the association remains (OR 2.19; CI 1.16–4.15) *p* = 0.016 (Table 3). Although there was an association between death and CC (RR 1.47; CI 95% 1.04–2.06) *p* = 0.027 (Figure 2), it disappeared after adjusting for sex, age, and APACHE II (Table 4). All of these analyses were also performed with CC without adjustment for BMI (Supplementary Data), and there were no significant results either.

## 4. Discussion

The main finding of this retrospective study was that in older adults with COVID-19, reduced CC is not associated with a systemic inflammatory profile, severity, or mortality markers but rather with nutritional status. Our study shows that patients with reduced CC are more likely to have greater nutritional risk and malnutrition. Observing that patients with reduced CC had fewer days of hospitalization in the ICU, we noticed that they had a higher number of deaths, and when analyzing the association between CC and death, there was no association after adjustment.

The sarcopenia consensus suggests a cutoff point < 31 cm for men and women, as this value predicts worse functional performance and survival in older adults [14]. However, we chose to use a cutoff point of CC < 33 cm for women and <34 cm for men, as it has a greater ability to predict a decrease in muscle mass in older adults [17], as this reference was made from older adults from the population of Brazil, corresponding to the public of our work. Furthermore, since BMI has an influence on CC, we chose to adjust CC according to BMI value, reducing by 3, 7, or 12 cm for a BMI of 25–29.9, 30–39.9, and ≥40 kg/m^2^, respectively [24]. When stratifying our sample by reduced and normal CC, we observed that there was a significant difference between the groups for sex, which is why we chose to stratify the sample by sex and reduced and normal CC.

Some studies have shown that male patients are at greater risk for COVID-19, as well as for mortality outcomes [27,28]. In the stratification for gender and reduced and normal CC, BMI continued to be significant between groups. As expected, patients with reduced CC had a lower BMI than patients with normal CC, since BMI is used to assess obesity [29], and the greater the amount of adipose and intramuscular tissue in the calves, the greater the CC value [30]. Obesity is a limitation for CC assessment [31], and there is no evidence that BMI values in critically ill patients reflect body composition [16]. For this reason, in our statistical analysis, we used BMI as an adjustment factor, given its influence on CC, as well as gender and age.

Our study also demonstrated an association of urea creatinine ratio between reduced and normal CC groups, which continued only for men after stratification by sex. When the regression was performed, we observed that there was an association between UCR and CC; however, it was lost after adjusting for sex, age, and BMI. As UCR has been used as a biochemical marker of catabolism due to loss of skeletal muscle mass [7,32], we imagined that in the reduced CC group, there would be an increase in UCR, indicating greater catabolism, but there was no significance.

Other data that showed significance between the reduced and normal CC groups, which continued even after stratification by gender, were the number of days of hospitalization in the ICU, and in the reduced CC group, the length of stay in the ICU was shorter, but it was the group with the highest number of deaths. When we performed the Cox regression, we observed that in the crude model, there was an association between mortality and CC, but after adjusting for sex, age, and APACHE II, this association was lost.

Some studies have already shown that CC is a good indicator of mortality [33,34]. However, in the cohort study by Wijnhoven et al., 2010 [29], which evaluated 1667 older adult residents in a nursing home, no association was found between CC and mortality. This corroborates our study, where we also did not find this association. We emphasize, however, that our sample consisted of older adults with COVID-19. In this sense, as it is an acute and inflammatory disease with rapid evolution [35], which mostly affects patients with excess adiposity [36], the assessment of CC upon admission loses its potential.

Clinical parameters that assess the severity and mortality of critically ill patients [10,33,34], such as the Sequential Organ Failure Assessment (SOFA) score [37], Acute Physiology and Chronic Health Evaluation (APACHE) II score [21], and lactate [38] were also considered in our analyses. However, no association with CC was found. Assessing whether CC would have a good association with the SOFA score, which is performed every 24 h inside the ICU and assesses acute morbidity [37,39], would place CC as a good parameter to assess acute morbidity in older adults with COVID-19. We had the same thought regarding the APACHE II score, which assesses severity and mortality [21,40].

Regarding lactate, its increase is a sign of the hypoperfusion of organs and muscular tissue and is associated with mortality [38,41]. We believe that as COVID-19 leads to profound hypoxemia and intense struggle to breathe [42], it would be increased in the bloodstream, leading to the hypoperfusion of muscle tissue, and would be associated with reduced CC by leading skeletal muscles to greater contraction in respiratory distress syndrome [38], but our study did not show an association between CC with lactate, and there are no studies evaluating CC with clinical parameters of severity and mortality such as SOFA, APACHE, and lactate.

Currently, there is no validated instrument for assessing nutritional risk in critically ill patients; the most used are NRS and NUTRIC [42]. The NRS is validated for assessing the nutritional risk of critically ill patients [43], but it is not specific for critically ill patients, while NUTRIC was created for assessing the nutritional risk of critically ill patients [43]. When evaluating the nutritional risk of the patients in the study, using these nutritional screening instruments, we observed significance between the reduced and normal CC groups, both in the evaluation of all patients and after stratification by sex, with patients with reduced CC having higher nutritional risk when performing a logistic regression, noting that in the adjusted model, there was a loss of association between instruments and CC. This corroborates some studies that have shown that patients at nutritional risk assessed by the NRS and NUTRIC had lower CC values [44,45,46]. There are no studies evaluating CC and nutritional risk in older adults with COVID-19.

Patients who are at nutritional risk are evaluated through nutritional screening, evaluated by an assessment of nutritional status; there is still no specific instrument for this assessment in critically ill patients [42]. One of the instruments used in the hospital routine is the SGA, which evaluates patients’ history and physical examination [23]. When we evaluated the nutritional status of this study’s patients, we noticed an association between the reduced and normal CC groups, both in the evaluation of all patients and in the stratification by gender, with the CC group comprising a greater number of moderately malnourished patients than the normal CC group. And when performing a logistic regression, observing the association between SGA and CC, both in the crude and in the adjusted model, patients with reduced CC were more likely to be moderately malnourished than nourished patients. Older adults are more willing to lose muscle mass [14] and are at greater risk of malnutrition in the ICU [11]; easily accessible instruments such as CC are necessary in a hospital environment [14]. Our study demonstrates that CC can be a good tool for the nutritional assessment of older adults with COVID-19 admitted to the ICU. Our study was the first to evaluate the association between CC and SGA in older adults with COVID-19.

We also believed that as COVID-19 is a highly inflammatory and catabolic disease [11], reduced CC would be associated with inflammatory parameters, but we did not find any association. In the systematic review and meta-analysis by Malik et al. (2020), it was shown that severe cases of COVID-19 are associated with an elevated CRP and lymphocyte count compared with milder cases. CPR is an acute-phase protein, synthesized by the liver in response to IL-6. With viral infection, IL-6 production increases, along with macrophage activation syndrome, increasing CRP [47,48]. Highly inflamed individuals have increased nutritional needs, increasing catabolic stress [49]. We believe that there was no association between CC and inflammatory parameters because COVID-19 is an acute disease [35] and may have a low influence on the reduction in muscle mass at the initial moment of the disease.

Another indicator of systemic inflammation is the NLR [50], which is elevated in patients with COVID-19. The increase in NLR means an increase in neutrophils and/or a decrease in lymphocytes [47]. A feature of viral infections that is common in patients with COVID-19 is lymphopenia, due to the depletion of circulating T cells during the inflammatory response [51]. Neutrophilia, on the other hand, is associated with a hyperinflammatory state and an increase in cytokines, and as the infectious condition worsens, there is an increase in the number of neutrophils [52]. Silva et al. (2022) [53] evaluated the association between NLR and the risk of sarcopenia in patients with COVID-19 and found no association between the risk of sarcopenia and NLR, corroborating our results that show no association between a decrease in muscle mass and NLR.

Patients with COVID-19 have a high inflammatory profile, which inhibits erythropoiesis, decreasing the production of red cells [51], presenting erythrocytes incapable of responding to environmental variations in HB oxygen saturation and compromising the ability to transport and provide oxygen, thus having a low concentration of HB [52]. Alnor et al. (2021) [54] performed a case–control study comparing hematological parameters in hospitalized patients with COVID-19 and symptomatic patients without COVID-19 and showed lower leukocyte, lymphocyte, monocyte, eosinophil, and basophil counts in the COVID-19 group than in the COVID-19 group. The study by Ghazanfari et al. in 2021 [55] also showed a decrease in red cells in patients with COVID-19.

We evaluated patients with a high inflammatory profile and tried to assess the association of HT and HB with CC, since patients with COVID-19 have high inflammation that can lead to protein catabolism [50] and the decrease in red cells with consequent loss of muscle mass [56]. However, we found no association between low muscle mass and HT and HB.

Our study was the first to assess HT and HB with CC and the association between CC and markers of inflammation, severity, nutritional risk (NRS) and nutritional status (SGA), and mortality in older adults with COVID-19. As positive points, we worked with a large sample and investigated instruments that are easy to obtain and low-cost for clinical practice. Furthermore, we performed the analysis both with CC adjusted for BMI and without adjustment to evaluate the data obtained. Our research suggests that CC may not be a good indicator to assess inflammation and mortality in patients with COVID-19, as it is an acute disease with a high inflammatory profile. New studies with a multicenter approach can accurately portray a large representation of the world’s population under these conditions. But it can be a good marker of nutritional status.

On the other hand, this study has some limitations, the first of which is the fact that it was a retrospective study carried out in a single center, and we also did not assess the nutritional status of the patients prior to ICU admission, which prevented a more meaningful analysis.

## 5. Conclusions

In conclusion, reduced CC was not associated with markers of inflammation, severity, nutritional risk, or mortality in our sample of inflammation in patients hospitalized with COVID-19, but it was associated with nutritional status.

## Figures and Tables

**Table 1:** Profile of elderly patients hospitalized in ICU with COVID-19 according to CC classification.

Variables	Total Sample	CC Reduced (*n* = 176)	CC Normal (*n* = 32)	*p* ^1^
Age (years) ^2^	72 (66–81)	73 (66–82)	68 (64.5–71)	<0.001 *
Sex (*n*, %) ^2^				0.313
Male	113 (54)	93 (52)	20 (62)	
Female	95 (46)	83 (47)	12 (37)	
Hospital length of stay (days)	10 (6–15)	9 (6–15)	13 (8–19)	0.016 *
Comorbidities (*n*, %) ^2^				
Diabetes ^2^	69 (33)	62 (40)	7 (24)	0.105
Hypertension ^2^	124 (59)	106 (68)	18 (62)	0.505
Chronic obstructive pulmonary disease ^2^	30 (14)	25 (16)	5 (17)	0.882
Chronic kidney disease ^2^	23 (11)	19 (12)	4 (13)	0.819
Cardiopathy ^2^	39 (18)	34 (22)	5 (17)	0.570
Dementia ^3^	17 (8)	13 (8)	4 (13)	0.356
Hypothyroidism ^2^	17 (8)	15 (9)	2 (7)	0.365
Cancer ^2^	24 (11)	20 (13)	4 (14)	0.896
No comorbidities ^2^	19 (9)	14 (9)	5 (17)	0.182
Invasive mechanical ventilation (*n*, %) ^2^	90 (43)	75 (48)	15 (51)	0.741
Vasoactive drugs (*n*, %) ^2^	52 (25)	44 (28)	8 (27)	0.930
Sedation (*n*, %) ^2^	63 (30)	54 (34)	9 (31)	0.692
Hemodialysis (*n*, %) ^2^	18 (9)	15 (9.6)	3 (10.3)	0.912
Edema (*n*, %) ^2^	26 (12)	18 (11)	8 (27)	0.023 *
Weight (kg)	72 (±15.9)	70 (±15.2)	77.9 (±18.8)	0.084 *
Height (m)	1.63 (±0.1)	1.62 (± 0.1)	1.67 (±0.1)	0.021 *
Body mass index (kg/m^2^)	26.5 (22.5–30)	23.4 (23.2–24.8)	27.5 (24.6–30.3)	0.466
Calf circumference (cm)	29.7 (±3.8)	28.8 (±3.3)	34.3 (±2.7)	<0.001 *
Hemoglobin (g/dL)	12.6 (11–13)	12.7 (11–13.8)	12.3 (9.8 -13.8)	0.450
Hematocrit (%)	36.8 (±7.3)	37 (±7.1)	35.9 (±8.1)	0.233
Urea (mg/dL)	57 (36–95)	58 (36–96.5)	54 (31–86)	0.445
Creatinine (mg/dL)	1.1 (0.8–1.8)	1.2 (0.8–1.8)	1.1 (0.8–1.8)	0.993
Urea to creatinine ratio	44 (33.3–58.3)	45 (33.5–59.1)	40.5 (31.4–57.8)	0.173
Neutrophil (×10^9^/L)	10 (6–14)	10 (6–14)	10.5 (7–13)	0.888
Lymphocyte (×10^9^/L)	808 (539–1149)	808 (539–1166.5)	803 (496–1070)	0.825
Neutrophil lymphocyte ratio	11.1 (6–18)	11.1 (5.6–15.3)	10.6 (6.77–20.3)	0.609
C reactive protein (mg/dL)	12.9 (7–20.9)	18.4 (15.1–23)	13.6 (13.3–21.4)	0.456
Lactate (mg/L)	18.2 (14.8–23)	18.2 (18.7–21.8)	17.4 (18.1–25.2)	0.351
SOFA score	6 (2–8)	6 (2–8)	5 (4–8)	0.805
APACHE II score	15 (11–26)	15.5 (11–26)	16.5 (11–25.5)	0.792
NUTRIC ^1^	5 (4–6)	5.0 (4–6)	4.5 (3.5–6)	0.432
NRS ^1^	4 (3–5)	4 (4–5)	3 (3–5)	0.011 *
≥3 ^3^	203 (97)	174 (99)	29 (90)	0.005 *
<3 ^3^	5 (3)	2 (1)	3 (10)	
SGA ^1^				
Well-nourished	100 (48)	80 (46)	20 (66.6)	0.118
Undernourished	103 (49)	92 (52)	10 (31)	
Death (*n*, %) ^2^	137 (65)	117 (66)	20 (62)	0.662

Variables are described in median and interquartile range or (*n*) and percentage. ^1^ Mann–Whitney test. ^2^ Chi-square test. ^3^ Fisher exact test. CC: calf circumference, SOFA: Sequential Organ Failure Assessment, APACHE: Acute Physiology and Chronic Health Evaluation, NUTRIC: Nutrition Risk in Critically III, NRS: Nutritional Risk Screening, SGA: Subjective Global Assessment. * Significant *p*-value (<0.05).

**Table 2:** Profile of elderly patients hospitalized in ICU with COVID-19 according to CC classification stratified by sex.

Variables	Men			Women		
	CC Reduced (*n* = 98)	CC Normal (*n* = 15)	*p* ^1^	CC Reduced (*n* = 83)	CC Normal (*n* = 12)	*p* ^1^
Age (years)	74 (67–81)	69 (64–75)	0.150	72 (66–83)	65 (62.5–67.5)	0.003 *
Hospital length of stay (days)	9 (6–14)	12.0 (7–22)	0.087	10 (6–15)	15.5 (8.5–21.5)	0.069
Comorbidities (*n*, %) ^2^						
Diabetes ^2^	32 (38)	3 (15)	0.064	30 (42)	4 (40)	0.892
Hypertension ^2^	56 (66)	12 (63)	0.771	50 (70)	6 (60)	0.504
Chronic obstructive pulmonary disease ^3^	14 (16)	4 (21)	0.649	11 (15)	1 (10)	0.647
Chronic kidney disease ^3^	13 (15)	3 (15)	0.973	6 (8)	1 (10)	0.870
Cardiopathy ^2^	16 (19)	3 (15)	0.741	18 (25)	2 (20)	0.713
Dementia ^3^	8 (9)	4 (21)	0.157	5 (7)	0	0.384
Hypothyroidism ^3^	3 (3.5)	1 (5.2)	0.730	12 (16)	1 (10)	0.578
Cancer ^3^	9 (10)	4 (21)	0.220	11 (15)	0	0.181
No comorbidities ^3^	7 (8)	3 (15)	0.322	7 (9)	2 (20)	0.339
Invasive mechanical ventilation (*n*, %) ^2^	40 (47)	11 (57)	0.419	35 (49)	4 (40)	0.220
Vasoactive drugs (*n*, %) ^2^	24 (28)	7 (36)	0.478	20 (28)	1 (10)	0.418
Sedation (*n*, %) ^2^	29 (34)	8 (42)	0.534	25 (35)	1 (10)	0.110
Hemodialysis (*n*, %) ^3^	8 (9)	2 (10)	0.894	7 (9)	1 (10)	0.989
Edema (*n*, %) ^3^	5 (5)	5 (26)	0.007	13 (18)	3 (30)	0.385
Weight (kg)	69 (±14)	83 (±18)	0.089	72.2 (±16)	77 (±19)	0.480
Height (m)	1.68 (±0.1)	1.73 (±0.1)	0.046 *	1.56 (±0.1)	1.58 (±0.1)	0.069
Body mass index (kg/m^2^)	24.1 (20–27)	25.7 (24.4–29.8)	0.370	28.3 (25.2–33.8)	28.8 (27.8–33.1)	0.242
Calf circumference (cm)	29.2 (±3.2)	35.9 (±1.8)	<0.001 *	28.3 (±3.26)	34 (±0.9)	<0.001 *
Hemoglobin (g/dL)	12.9 (11.1–14.1)	13.2 (9.6–14.8)	0.752	12.5 (10.9–13.5)	11.7 (10.5–12.6)	0.217
Hematocrit (%)	37.3 (±7.5)	36 (±9.7)	0.449	36.7 (±6.6)	35 (±6.5)	0.305
Urea (mg/dL)	66 (43–107)	71 (41–119)	0.943	47 (28–83)	29.5 (25–43.5)	0.075
Creatinine (mg/dL)	1.3 (0.9–2.3)	1.5 (1–2.3)	0.634	1 (0.7–1.6)	0.8 (0.7–1.0)	0.395
Urea to creatinine ratio	48.2 (35.4–61)	41 (32–59.2)	0.676	42.5 (31.7–20.8)	31.8 (26.4–42)	0.062
Neutrophil(×10^9^/L)	10.0 (8–14)	12 (8–14)	0.874	8 (5–14)	8.0 (8.4–11.3)	0.621
Lymphocyte(×10^9^/L)	783 (538–1124)	670 (388–984)	0.500	867 (553–1226)	915 (700–1487)	0.452
Neutrophil lymphocyte ratio	11.8 (7.28–18.4)	18.2 (7.25–30.6)	0.479	7.8 (4.5–15)	7.5 (6–12.5)	0.805
C reactive protein (mg/dL)	12.8 (7.4–1.8)	12 (6.3–22)	0.582	12.6 (5.9–20.8)	15.6 (8.7–21)	0.753
Lactate(mmol/L)	19.4 (16.7–24.6)	18.6 (13–25.6)	0.187	16.8 (13–20.7)	16.4 (12.1–25.2)	0.788
SOFA score	6.0 (3.5–8.0)	6 (4–8)	0.975	4 (2–8)	4.5 (3–7)	0.817
APACHE II score	17.5 (12–29)	24 (15–29)	0.457	13.0 (10–24)	10.5 (7–18.5)	0.076
NUTRIC ^2^	5 (4–6)	5 (4–6)	0.790	4 (3–6)	3.5 (2.5–5.5)	0.071
NRS ^2^	4 (4–5)	4 (3–6)	0.372	4 (3–5)	3 (3–4)	<0.001 *
≥3 ^3^	91 (97)	15 (100)	1.000	83 (100)	9 (75)	<0.001 *
<3 ^3^	2 (2.1)	0		0	3 (25)	
SGA ^2^						
Well-nourished	37 (40)	10 (66)	0.720	43 (52)	10 (83)	0.016 *
Undernourished	54 (55)	10 (66)		39 (47)	0	
Death (*n*, %) ^2^	60(64)	15 (100)	0.488	57 (68)	5 (41)	0.066

Variables are described in median and interquartile range or (*n*) and percentage and mean ± standard deviation from the mean. ^1^ Obtained by the analysis of t Student unpaired test or Mann-Whitney test. ^2^ Chi-square ^3^ Fisher exact test. APACHE: Acute Physiology and Chronic Health Evaluation, NUTRIC: Nutrition Risk in Critically III, NRS: Nutritional Risk Screening, SGA: Subjective Global Assessment. * Significant *p*-value (<0.05).

**Table 3:** Association between CC and biochemical and clinical characteristics in elderly patients admitted to ICU with COVID-19.

	Model 1		Model 2	
Variables	OR (95% CI)	*p*	OR (95% CI)	***p***
Hemoglobin (g/dL)	1.06 (0.91; 1.24)	0.396	1.05 (0.90; 1.23)	0.475
Hematocrit (%)	1.03 (0.98; 1.08)	0.224	1.02 (0.97; 1.07)	0.310
Urea (mg/dL)	1.00 (1.00; 1.01)	0.233	1.00 (0.99; 1.01)	0.229
Creatinine (mg/dL)	0.98 (0.89; 1.08)	0.799	0.91 (0.76; 1.10)	0.359
Urea to creatinine ratio	1.01 (0.99; 1.03)	0.153	1.01 (0.99; 1.03)	0.194
Neutrophil(×10^9^/L)	1.01 (0.93; 1.09)	0.721	1.01 (0.89; 1.15)	0.789
Lymphocyte(×10^9^/L)	1.00 (1.00; 1.00)	0.782	0.99 (0.99; 1.00)	0.976
Neutrophil lymphocyte ratio	0.98 (0.96; 1.00)	0.214	0.98 (0.96; 1.01)	0.295
C reactive protein (mg/dL)	0.99 (0.95; 1.02)	0.624	0.99 (0.95;1.03)	0.764
Lactate (mmol/L)	1.01 (0.97; 1.06)	0.390	1.02 (0.97; 1.07)	0.403
Diabetes	2.09 (0.84; 5.20)	0.111	2.06 (0.81; 5.25)	0.127
Hypertension	1.32 (0.58; 3.01)	0.506	0.96 (0.39; 2.31)	0.928
Chronic obstructive pulmonary disease	0.92 (0.32; 2.64)	0.882	0.66 (0.21; 2.06)	0.485
Chronic kidney disease	0.87 (0.27; 2.78)	0.819	1.00 (0.30; 3.36)	0.994
Cardiopathy	1.34 (047; 3.80)	0.571	1.01 (0.34; 2.99)	0.973
Dementia	0.57 (0.17; 1.89)	0.361	0.33 (0.08; 1.28)	0.111
Hypothyroidism	1.44 (0.31; 6.69)	0.637	0.94 (0.18; 4.75)	0.942
Cancer	0.92 (0.29; 2.93)	0.896	1.04 (0.32; 3.43)	0.936
No comorbidities	2.09 (0.69; 6.36)	0.190	1.45 (0.45; 4.66)	0.524
Invasive mechanical ventilation	0.87 (0.39; 1.93)	0.742	0.98 (0.41; 2.32)	0.975
Vasoactive drugs	1.04 (0.42; 2.52)	0.930	1.13 (0.41; 3.08)	0.806
Sedation	1.18 (0.50; 2.78)	0.692	1.25 (0.48; 3.22)	0.642
Hemodialysis	0.92 (0.25; 3.43)	0.912	1.09 (0.27; 4.31)	0.898
Edema (*n*, %)	0.34 (0.13; 0.89)	0.028 *	0.26 (0.09; 0.74)	0.012 *
SOFA score	1.00 (0.89; 1.11)	0.988	1.01 (0.86; 1.18)	0.881
NUTRIC	1.12 (0.89; 1.41)	0.320	1.11 (0.72; 1.73)	0.619
NRS (continuous)	1.49 (1.06; 2.10)	0.021 *	1.45 (1.01; 2.10)	0.043 *
NRS (≥3 vs. <3)	0.11 (0.01; 0.69)	0.019 *	2.11 (0.89; 5.02)	0.089
SGA (well-nourished vs. undernourished)	2.32 (1.02; 5.25)	0.043 *	2.19 (1.16; 4.15)	0.016 *
Hospital length of stay (days)	1.18 (054; 2.59)	0.663	1.10 (0.46; 2.59)	0.827

Model 1: Crude model; Model 2: adjusted model by sex, age, and APACHE II. APACHE: Acute Physiology and Chronic Health Evaluation, NUTRIC: Nutrition Risk in Critically III, NRS: Nutritional Risk Screening, SGA: Subjective Global Assessment. * Significant *p*-value (<0.05).

**Table 4:** Survival model stratified by CC in patients admitted to ICU with COVID-19.

	Model 1		Model 2	
Variables	OR (95% CI)	*p*	OR (95% CI)	*p*
CC (reduced vs. normal)	1.47 (1.04; 2.06)	0.027 *	1.31 (0.86; 1.98)	0.204

Crude Cox model. Adjusted model by sex, age, and APACHE II. * Significant *p* value (<0.05).

## Data Availability

Data is unavailable due to privacy and ethical restrictions.

## Supplementary Materials

The following supporting information can be downloaded at https://www.mdpi.com/article/10.3390/diseases12050097/s1, Table S1: Profile of elderly patients hospitalized in ICU with COVID-19 according to CC classification; Table S2: Profile of elderly patients hospitalized in ICU with COVID-19 according to CC classification stratified by sex; Table S3: Association between CC and biochemical and clinical characteristics in elderly patients admitted to ICU with COVID-19; Table S4: Survival model stratified by CC in patients admitted to ICU with COVID-19; Figure S1: Pearson correlation graph between BMI and CC of patients admitted to the ICU with COVID-19; Figure S2: Survival curve of patients admitted at ICU with COVID-19 by calf circumference classification.

## References

[1] Santer D., Schneider N., de Carvalho Y.S.S., de Souza Bortolini R.V., Silva F.M., Franken D.L., da Silva Fink J. (2023). The association between reduced calf and mid-arm circumferences and ICU mortality in critically ill COVID-19 patients. Clin. Nutr. ESPEN.

[2] Osuna-Padilla I., Rodríguez-Moguel N., Rodríguez-Llamazares S., Orsso C., Prado C., Ríos-Ayala M., Villanueva-Camacho O., Aguilar-Vargas A., Pensado-Piedra L., Juárez-Hernández F. (2022). Low muscle mass in COVID-19 critically-ill patients: Prognostic significance and surrogate markers for assessment. Clin. Nutr..

[3] Piotrowicz K., Gąsowski J., Michel J.-P., Veronese N. (2021). Post-COVID-19 acute sarcopenia: Physiopathology and management. Aging Clin. Exp. Res..

[4] Qin C., Zhou L., Hu Z., Zhang S., Yang S., Tao Y., Xie C., Ma K., Shang K., Wang W. (2020). Dysregulation of Immune Response in Patients with Coronavirus 2019 (COVID-19) in Wuhan, China. Clin. Infect. Dis..

[5] Padilha D.M.H., Mendes M.C.S., Lascala F., Silveira M.N., Pozzuto L., Santos L.A.O., Guerra L.D., Moreira R.C.L., Branbilla S.R., Junior A.D.C. (2022). Low skeletal muscle radiodensity and neutrophil-to-lymphocyte ratio as predictors of poor outcome in patients with COVID-19. Sci. Rep..

[6] Gil S., Filho W.J., Shinjo S.K., Ferriolli E., Busse A.L., Avelino-Silva T.J., Longobardi I., de Oliveira Júnior G.N., Swinton P., Gualano B. (2021). Muscle strength and muscle mass as predictors of hospital length of stay in patients with moderate to severe COVID-19: A prospective observational study. J. Cachexia Sarcopenia Muscle.

[7] Flower L., Haines R.W., McNelly A., Bear D.E., Koelfat K., Damink S.O., Hart N., Montgomery H., Prowle J.R., Puthucheary Z. (2021). Effect of intermittent or continuous feeding and amino acid concentration on urea-to-creatinine ratio in critical illness. J. Parenter. Enter. Nutr..

[8] Ali A.M., Kunugi H. (2021). Skeletal Muscle Damage in COVID-19: A Call for Action. Medicina.

[9] Zhang X.-M., Wu X., Ma Y., Zhu C., Cao J., Liu G., Li F.-F., Cheng A.S. (2021). Comparing the Performance of Calf Circumference, Albumin, and BMI for Predicting Mortality in Immobile Patients. Risk Manag. Healthc. Policy.

[10] Zhao H., Davies R., Ma D. (2021). Potential therapeutic value of dexmedetomidine in COVID-19 patients admitted to ICU. Br. J. Anaesth..

[11] Barazzoni R., Bischoff S.C., Breda J., Wickramasinghe K., Krznaric Z., Nitzan D., Pirlich M., Singer P. (2020). ESPEN expert statements and practical guidance for nutritional management of individuals with SARS-CoV-2 infection. Clin. Nutr..

[12] Mechanick J.I., Carbone S., Dickerson R.N., Hernandez B.J.D., Hurt R.T., Irving S.Y., Li D., McCarthy M.S., Mogensen K.M., Gautier J.B.O. (2021). Clinical Nutrition Research and the COVID-19 Pandemic: A Scoping Review of the ASPEN COVID-19 Task Force on Nutrition Research. J. Parenter. Enter. Nutr..

[13] Bahat G. (2021). Measuring calf circumference: A practical tool to predict skeletal muscle mass via adjustment with BMI. Am. J. Clin. Nutr..

[14] Cruz-Jentoft A.J., Bahat G., Bauer J., Boirie Y., Bruyère O., Cederholm T., Cooper C., Landi F., Rolland Y., Sayer A.A. (2019). Sarcopenia: Revised European consensus on definition and diagnosis. Age Ageing.

[15] Bunnell K.M., Thaweethai T., Buckless C., Shinnick D.J., Torriani M., Foulkes A.S., Bredella M.A. (2021). Body composition predictors of outcome in patients with COVID-19. Int. J. Obes..

[16] Poros B., Becker-Pennrich A.S., Sabel B., Stemmler H.J., Wassilowsky D., Weig T., Hinske L.C., Zwissler B., Ricke J., Hoechter D.J. (2021). Anthropometric analysis of body habitus and outcomes in critically ill COVID-19 patients. Obes. Med..

[17] Pagotto V., Santos KF dos Malaquias S.G., Bachion M.M., Silveira E.A. (2018). Calf circumference: Clinical validation for evaluation of muscle mass in the elderly. Rev. Bras. Enferm..

[18] Alsharif W., Qurashi A. (2021). Effectiveness of COVID-19 diagnosis and management tools: A review. Radiography.

[19] Petersen S.W. (2016). Advanced Health Assessment and Diagnostic Reasoning.

[20] Vincent J.-L., Taccone F.S. (2020). Understanding pathways to death in patients with COVID-19. Lancet Respir. Med..

[21] Knaus W.A., Draper E.A., Wagner D.P., Zimmerman J.E. (1985). APACHE II: A severity of disease classification system. Crit. Care Med..

[22] Heyland D.K., Dhaliwal R., Jiang X., Day A.G. (2011). Identifying critically ill patients who benefit the most from nutrition therapy: The development and initial validation of a novel risk assessment tool. Crit. Care.

[23] Sousa I.M., Fayh A.P.T., Lima J., Gonzalez M.C., Prado C.M., Silva F.M. (2023). Low calf circumference adjusted for body mass index is associated with prolonged hospital stay. Am. J. Clin. Nutr..

[24] Bolayir B., Arik G., Yeşil Y., Kuyumcu M.E., Varan H.D., Kara Ö., Güngör A.E., Yavuz B.B., Cankurtaran M., Halil M.G. (2019). Validation of Nutritional Risk Screening-2002 in a Hospitalized Adult Population. Nutr. Clin. Pract..

[25] Duerksen D.R., Yeo T.A., Siemens J.L., O’Connor M.P. (2000). The validity and reproducibility of clinical assessment of nutritional status in the elderly. Nutrition.

[26] Lipschitz D.A. (1994). Screening for nutritional status in the elderly. Prim. Care Clin. Off. Pract..

[27] Bienvenu L.A., Noonan J., Wang X., Peter K. (2020). Higher mortality of COVID-19 in males: Sex differences in immune response and cardiovascular comorbidities. Cardiovasc. Res..

[28] Feng H., Gan C.C.R., Leiva D., Zhang B.L., Davies S.E. (2022). COVID-19, sex, and gender in China: A scoping review. Glob. Health.

[29] Wijnhoven H.A., van Bokhorst-de van der Schueren M.A., Heymans M.W., de Vet H.C., Kruizenga H.M., Twisk J.W., Visser M. (2010). Low Mid-Upper Arm Circumference, Calf Circumference, and Body Mass Index and Mortality in Older Persons. J. Gerontol. Ser. A Biol. Sci. Med. Sci..

[30] Hilton T.N., Tuttle L.J., Bohnert K.L., Mueller M.J., Sinacore D.R. (2008). Excessive Adipose Tissue Infiltration in Skeletal Muscle in Individuals With Obesity, Diabetes Mellitus, and Peripheral Neuropathy: Association With Performance and Function. Phys. Ther..

[31] Lim W.S., Lim J.P., Chew J., Tan A.W.K. (2020). Calf Circumference as a Case-Finding Tool for Sarcopenia: Influence of Obesity on Diagnostic Performance. J. Am. Med. Dir. Assoc..

[32] Gunst J., Kashani K.B., Hermans G. (2019). The urea-creatinine ratio as a novel biomarker of critical illness-associated catabolism. Intensive Care Med..

[33] Wei J., Jiao J., Chen C.-L., Tao W.-Y., Ying Y.-J., Zhang W.-W., Wu X.-J., Zhang X.-M. (2022). The association between low calf circumference and mortality: A systematic review and meta-analysis. Eur. Geriatr. Med..

[34] Fernandes D.P.S., Juvanhol L.L., Lozano M., Ribeiro A.Q. (2022). Calf circumference is an independent predictor of mortality in older adults: An approach with generalized additive models. Nutr. Clin. Pract..

[35] Merad M., Blish C.A., Sallusto F., Iwasaki A. (2022). The immunology and immunopathology of COVID-19. Science.

[36] Colleluori G., Graciotti L., Pesaresi M., Di Vincenzo A., Perugini J., Di Mercurio E., Caucci S., Bagnarelli P., Zingaretti C.M., Nisoli E. (2022). Visceral fat inflammation and fat embolism are associated with lung’s lipidic hyaline membranes in subjects with COVID-19. Int. J. Obes..

[37] Ferreira F.L. (2001). Serial Evaluation of the SOFA Score to Predict Outcome in Critically Ill Patients. JAMA.

[38] Gupta G.S. (2022). The Lactate and the Lactate Dehydrogenase in Inflammatory Diseases and Major Risk Factors in COVID-19 Patients. Inflammation.

[39] Lambden S., Laterre P.F., Levy M.M., Francois B. (2019). The SOFA score—Development, utility and challenges of accurate assessment in clinical trials. Crit. Care.

[40] Naved S.A., Siddiqui S., Khan F.H. (2011). APACHE-II Score Correlation with Mortality and Length of Stay in an Intensive Care Unit. J. Coll. Physicians Surg. Pak..

[41] Iepsen U.W., Plovsing R.R., Tjelle K., Foss N.B., Meyhoff C.S., Ryrsø C.K., Berg R.M.G., Secher N.H. (2022). The role of lactate in sepsis and COVID-19: Perspective from contracting skeletal muscle metabolism. Exp. Physiol..

[42] Singer P., Blaser A.R., Berger M.M., Alhazzani W., Calder P.C., Casaer M.P., Hiesmayr M., Mayer K., Montejo J.C., Pichard C. (2019). ESPEN guideline on clinical nutrition in the intensive care unit. Clin. Nutr..

[43] Kondrup J. (2003). Nutritional risk screening (NRS 2002): A new method based on an analysis of controlled clinical trials. Clin. Nutr..

[44] Leandro-Merhi V.A., Costa C.L., Saragiotto L., de Aquino J.L.B. (2019). Nutritional indicators of malnutrition in hospitalized patients. Arq. Gastroenterol..

[45] Zhang X.Y., Zhang X.L., Zhu Y.X., Jun T.A.O., Zhang Z., Zhang Y., Wang Y.Y., Ke Y.Y., Ren C.X., Jun X.U. (2019). Low Calf Circumference Predicts Nutritional Risks in Hospitalized Patients Aged More Than 80 Years. Biomed. Environ. Sci..

[46] Lee Z.Y., Hasan M.S., Day A.G., Ng C.C., Ong S.P., Yap C.S.L., Engkasan J.P., Barakatun-Nisak M.Y., Heyland D.K. (2022). Initial development and validation of a novel nutrition risk, sarcopenia, and frailty assessment tool in mechanically ventilated critically ill patients: The NUTRIC-SF score. J. Parenter. Enter. Nutr..

[47] Paces J., Strizova Z., Smrz D., Cerny J. (2020). COVID-19 and the Immune System. Physiol. Res..

[48] Smilowitz N.R., Kunichoff D., Garshick M., Shah B., Pillinger M., Hochman J.S., Berger J.S. (2021). C-reactive protein and clinical outcomes in patients with COVID-19. Eur. Heart J..

[49] Hinkelmann J.V., de Oliveira N.A., Marcato D.F., Costa A.R.R.O., Ferreira A.M., Tomaz M., Rodrigues T.J., Mendes A.P. (2022). Nutritional support protocol for patients with COVID-19. Clin. Nutr. ESPEN.

[50] Borges T.C., Gomes T.L.N., Pimentel G.D. (2020). Sarcopenia as a predictor of nutritional status and comorbidities in hospitalized patients with cancer: A cross-sectional study. Nutrition.

[51] Karimi Shahri M., Niazkar H.R., Rad F. (2021). COVID-19 and hematology findings based on the current evidences: A puzzle with many missing pieces. Int. J. Lab. Hematol..

[52] Palladino M. (2021). Complete blood count alterations in COVID-19 patients: A narrative review. Biochem. Medica.

[53] Silva J., Giglio B.M., Lobo P.C.B., Araújo V.A., Pimentel G.D. (2022). Neutrophil-to-lymphocyte ratio is not associated with risk of sarcopenia in elderly COVID-19 patients. Rev. Española Geriatría Gerontol..

[54] Alnor A., Sandberg M.B., Toftanes B.E., Vinholt P.J. (2021). Platelet parameters and leukocyte morphology is altered in COVID-19 patients compared to non-COVID-19 patients with similar symptomatology. Scand. J. Clin. Lab. Investig..

[55] Ghazanfari T., Salehi M.R., Namaki S., Arabkheradmand J., Rostamian A., Chenary M.R., Ghaffarpour S., Ardestani S.K., Edalatifard M., Naghizadeh M.M. (2021). Interpretation of Hematological, Biochemical, and Immunological Findings of COVID-19 Disease: Biomarkers Associated with Severity and Mortality. Iran. J. Allergy Asthma Immunol..

[56] Kim K.M., Lim S., Oh T.J., Moon J.H., Choi S.H., Lim J.Y., Kim K.W., Park K.S., Jang H.C. (2018). Longitudinal Changes in Muscle Mass and Strength, and Bone Mass in Older Adults: Gender-Specific Associations Between Muscle and Bone Losses. J. Gerontol. Ser. A.

