# Supplementary material for: Association of Calf Circumference with Clinical and Biochemical Markers in Older Adults with COVID-19 Admitted at Intensive Care Unit: A Retrospective Cross-Sectional Study
Source: Diseases. 2024 May 8;12(5):97. doi: 10.3390/diseases12050097 (PMC11119336; doi:10.3390/diseases12050097)
Supplement: Supplementary file 1 [file diseases-12-00097-s001.zip › diseases-2959176-supplementary.pdf]

Supplementary Data

Table 1. Profile of elderly patients hospitalized at ICU with COVID-19 according to CC classification.

	Total sample	CC Reduced (n= 120)	CC Normal (n= 88)	p[1]
Age (years)[2]	72 (66 - 81)	76 (67 - 84)	70 (65 - 75)	**<0.001***
Sex (n, %)[2]				
Male	113 (54)	76 (63)	37 (63)	**0.002***
Female	95 (46)	44 (37)	51 (37)	
Hospital Length of Stay (days)	10 (6 - 15)	10 (6 - 14)	13 (7 - 17)	**0.019***
Comorbidities (n, %)[2]				
Diabetes[2]	69 (33)	43 (39)	26 (34)	0.510
Hypertension[2]	124 (59)	69 (63)	55 (63)	0.154
Chronic obstructive pulmonary disease[2]	30 (14)	22 (20)	8 (10)	0.086
Chronic kidney disease[2]	23 (11)	15 (13)	8 (10)	0.533
Cardiopathy[2]	39 (18)	22 (20)	17 (22)	0.685
Dementia[3]	17 (8)	13 (12)	4 (5)	0.129
Hypothyroidism[2]	17 (8)	8 (7)	9 (12)	0.283
Cancer[2]	24 (11)	15 (13)	9 (12)	0.727
No comorbidities[2]	19 (9)	11 (14)	8 (7)	0.108
Invasive Mechanical Ventilation (n, %)[2]	90 (43)	51 (46)	39 (52)	0.487
Vasoactivedrugs (n, %)[2]	52 (25)	30 (27)	22 (30)	0.789
Sedation (n, %)[2]	63 (30)	36 (33)	27 (36)	0.676
Hemodialysis (n, %)[2]	18 (9)	11 (10)	7 (9.3)	0.865
Edema (n, %)[2]	26 (12)	11 (10)	15 (20)	0.058
Weight (kg)	72 (± 15.9)	64.2 (±12.2)	82 (±14.3)	**<0.001***
Height (m)	1.63 (± 0.1)	1.62 (± 0.1)	1.64 (± 0.1)	**0.345**
Body mass index (kg/m²)	26.5 (22.5 - 30)	24.3 (21 - 27.2)	30.6 (27 - 34)	**<0.001***
Calf circumference (cm)	29.7 (± 3.8)	29 (± 3)	37.2 (± 3)	**<0.001***
Hemoglobin (g/dL)	12.6 (11 - 13)	12.4 (11 - 14)	12.2 (11 - 13.7)	0.556
Hematocrit (%)	36.8 (± 7.3)	37 (± 7.3)	36.4 (± 7.2)	0.526

Urea(mg/dL)	57 (36 - 95)	82.3 (40 - 102)	63.5 (30 - 80)	**0.025***
Creatinine(mg/dL)	1.1 (0.8 - 1.8)	2.1 (0.8 - 1.75)	1.76 (0.8 - 1.8)	0.778
Urea to creatinine ratio	44 (33.3 - 58.3)	52.7 (35 - 64.3)	41.8 (30.6 - 48.5)	**<0.001***
Neutrophil($x10^9$/L)	10 (6 - 14)	10.2 (7 - 13)	10.4 (6 - 14)	0.598
Lymphocyte($x10^9$/L)	808 (539 - 1149)	1042 (530 - 1123)	1045 (553 - 1362)	0.480
Neutrophil lymphocyte ratio	11.1 (6 - 18)	14.8 (5.5 - 15.3)	14.5 (6.3 - 18)	0.767
C reactive protein(mg/dL)	12.9 (7 - 20.9)	15 (7 - 22)	14 (7 - 20.8)	0.691
Lactate(mg/L)	18.2 (14.8 - 23)	20.4 (16 - 21.3)	21.7 (13.7 - 25)	0.596
SOFA score	6 (2 - 8)	5.2 (2 - 8)	5.6 (2 - 8)	0.279
APACHE II score	15 (11 - 26)	18.5 (11 - 25)	19.1 (11 - 26)	0.609
NUTRIC[1]	5 (4 - 6)	5 (4 - 6)	4.8 (3 - 6)	0.544
NRS[1]	4 (3 - 5)	4.5 (4 - 5)	4 (3 - 5)	**<0.001***
≥3[3]	203 (97)	118 (98)	85 (96)	
<3[3]	5 (3)	2 (1.6)	3 (3.4)	0.418
SGA[1]				
Well-nourished	100 (48)	38 (32)	62 (74)	**<0.001***
Undernourished	103 (49)	81 (67)	22 (25)	
Death (n, %)[2]	137 (65)	79 (65)	58 (65)	0.991

Variables are described in median and interquartile range or (n) and percentage[1]Mann-whitney test. [2]Chi-square test. [3] Fisher exact test. CC: calf circumference, SOFA: Sequential Organ Failure Assessment, APACHE: Acute Physiology and Chronic Health Evaluation, NUTRIC: Nutrition Risk in Critically Ill, NRS: Nutritional Risk Screening, SGA: Subjective Global Assessment *Significant p value (<0.05).

Table 2. Profile of elderly patients hospitalized at ICU with COVID-19 according to CC classification stratified by sex.

	**Men**			**Women**		
	**CC reduced (n= 76)**	**CC normal (n= 37)**	**p[1]**	**CC reduced (n= 44)**	**CC normal (n= 51)**	**p[1]**
Age (years)	75.5 (69 - 84)	69 (64- 75)	**0.007***	75 (67 - 84)	67 (64 - 73)	**0.002***
Hospital Length of Stay (days)	8 (6 - 13)	13 (7 - 19)	**0.003***	10 (5 - 15)	12 (7 - 17)	0.334
Comorbidities (n, %)[2]						
Diabetes[2]	26 (37)	9 (26)	0.259	17 (42)	17 (41)	0.925
Hypertension[2]	45 (65)	23 (67)	0.807	24 (60)	32 (78)	0.079
Chronic obstructive pulmonary disease[3]	15 (21)	3 (8.8)	0.105	7 (17)	5 (12)	0.502

Chronic kidney disease³	13 (17)	4 (11)	0.458	3 (7.5)	4 (9.7)	0.718
Cardiopathy²	11 (16)	3 (23)	0.351	11 (27)	9 (22)	0.563
Dementia³	9 (13)	3 (8.8)	0.530	4 (10)	1 (2)	0.157
Hypothyroidism³	2 (2.9)	2 (5.8)	0.461	6 (15)	7 (17)	0.799
Cancer³	9 (13)	4 (11)	0.854	6 (15)	5 (12)	0.713
No comorbidities³	4 (5.8)	6 (17.6)	0.056	4 (10)	5 (12)	0.753
Invasive Mechanical Ventilation (n, %)²	33 (47)	18 (53)	0.625	18 (45)	21 (51)	0.575
Vasoactive drugs (n, %)²	21 (30)	10 (29)	0.731	9 (22)	12 (29)	0.487
Sedation (n, %)²	24 (34)	13 (38)	0.915	12 (30)	14 (34)	0.689
Hemodialysis (n, %)³	2 (5.8)	8 (11.5)	0.357	3 (7.5)	5 (12)	0.479
Edema (n, %)³	5 (7.25)	5 (14.7)	0.229	6 (15)	10 (24)	0.289
Weight (kg)	65 (±11)	85 (±14.2)	**<0.001***	62.6 (± 12.6)	82 (± 14)	**<0.001***
Height (m)	1.66 (± 0.1)	1.73 (± 0.1)	**<0.001***	1.56 (± 0.1)	1.58 (± 0.1)	**0.010***
Body mass index (kg/m^2)	23 (20 - 25)	28.3 (25 - 30.8)	**<0.001***	26 (22.3 - 28.3)	32.4 (28 - 36)	**<0.001***
Calf circumference (cm)	29.6 (± 3.0)	37.69 (± 3.2)	**<0.001***	29 (± 3)	37 (± 3)	**<0.001***
Hemoglobin (g/dL)	12.9 (11.1 - 14)	13.4 (10.8 - 15)	0.931	12.6 (11 - 14)	12.1(11 - 13.2)	0.632
Hematocrit (%)	37.4 (± 7.3)	36.5 (± 9)	0.652	37 (± 7.3)	36 (± 5.9)	0.802
Urea (mg/dL)	69 (44 - 113)	60 (38 - 110)	0.188	50 (29 - 73)	40 (27 - 70)	0.540
Creatinine (mg/dL)	1.3 (0.9 - 2.3)	1.5 (1 - 2.3)	0.199	1 (0.7 - 1.5)	1 (0.8 - 1.6)	0.770
Urea to creatinine ratio	50.4 (37.4 - 64.3)	40 (33 - 53)	**<0.001***	44.2 (32 - 64)	38 (30 - 48)	0.401
Neutrophil($x10^9/L$)	10.0 (8 - 13)	10 (8 - 14)	0.584	8 (5 - 14)	10 (5 - 15)	0.513
Lymphocyte($x10^9/L$)	671 (480 - 1084)	808 (540 - 1265)	0.164	886 (650 - 1218)	782 (526 - 1352)	0.384
Neutrophil lymphocyte ratio	12.7 (7.28 - 18.4)	11.2 (7.25 - 18.6)	0.832	6.6 (4.5 - 14)	8.6 (5 - 15)	0.065
C reactive protein (mg/dL)	12.8 (7.3 - 21.6)	12 (7.4 - 22)	0.631	11.1 (6.3 - 22.2)	14.7 (6.4 - 20.8)	0.899
Lactate(mmol/L)	19.4 (16.8 - 24.6)	18.6 (13.8 - 25)	0.318	16.5 (13 - 18.4)	17.4 (12.6 - 25.2)	0.405
SOFA score	6.0 (4 - 8.0)	6 (3 - 8)	0.420	3.5 (2 - 7)	6 (2 - 8)	0.192
APACHE II score	18 (12 - 29)	22 (12 - 29)	0.533	13.0 (10 - 21)	15 (10 - 25)	0.248
NUTRIC²	5 (4 - 6)	5 (4 - 6)	0.865	4 (3 - 6)	4 (3 - 6)	0.768
NRS²	4 (4 - 6)	4 (3 - 5)	0.094	4 (3 - 5)	3.6 (3 - 4)	**0.007***
≥3³	74 (97)	37 (100)	0.319	44 (100)	48 (94)	0.102
<3³	2 (2.6)	0		0	3 (5)	
SGA²						
Well-nourished	23 (30)	24 (66)		15 (34)	38 (79)	

Undernourished	52 (69)	12 (33)	**<0.001***	29 (65)	10 (20)	**<0.001***
Death (n, %)^2	52 (68)	23 (62)	0.509	27 (61)	35 (68)	0.458

Variables are described in median and interquartile range or (n) and percentage and mean ± standard deviation from the mean.[1]Obteined by the analysis of t Student unpaired test or Mann-whitney test. [2]Chi-square [3] Fisher exact test.APACHE: Acute Physiology and Chronic Health Evaluation, NUTRIC: Nutrition Risk in Critically III, NRS: Nutritional Risk Screening, SGA: Subjective Global Assessment. *Significant p value (<0.05).

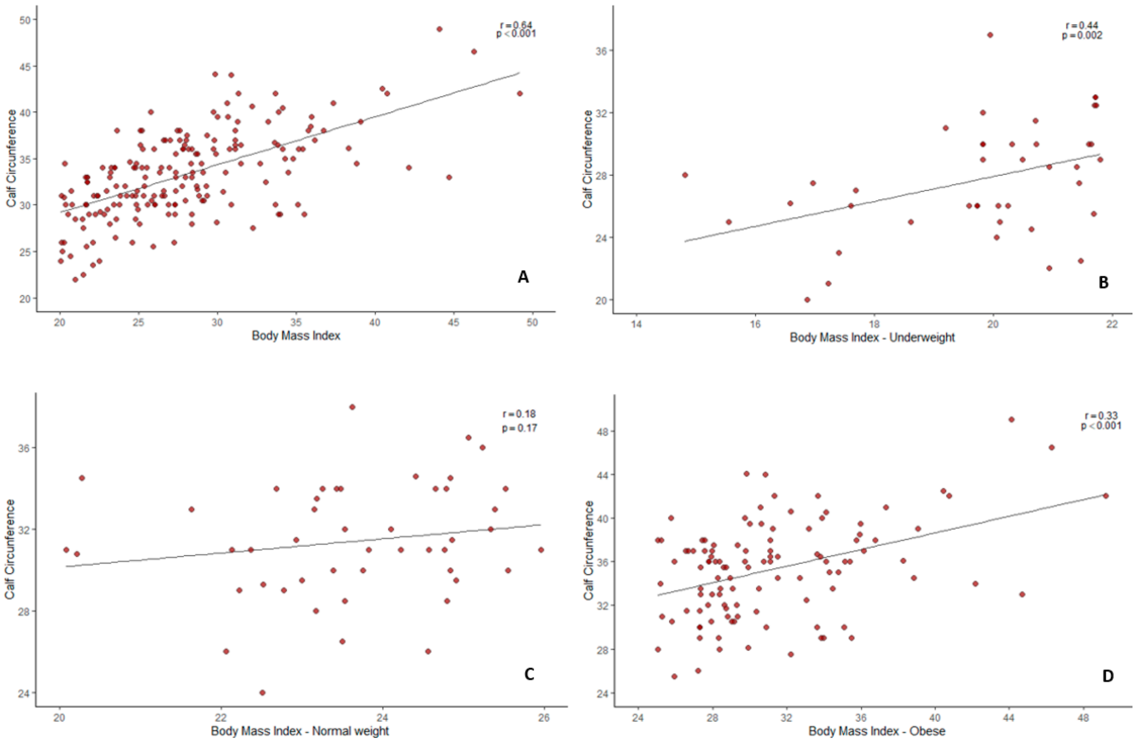

Figure 1. A: Pearson correlation graph between BMI and CC of patients admitted to the ICU with covid-19. **B**: Pearson correlation chart between underweight BMI and CC of patients admitted to the ICU with covid-19. **C**: Pearson correlation chart between normal BMI and CC of patients admitted to the ICU with covid-19. **D**: Pearson correlation chart between BMI overweight/obesity and CC of patients admitted to the ICU with covid-19.

Table 3. Association between CC and biochemical and clinical characteristics in elderly patients admitted at ICU with COVID-19.

	Model 1		Model 2	
**Variables**	**OR (95% CI)**	**p**	**OR (95% CI)**	**P**
Hemoglobin (g/dL)	1.02 (0.91; 1.15)	0.632	0.99 (0.87; 1.12)	0.881
Hematocrit (%)	1.01 (0.97; 1.05)	0.547	1.00 (0.96; 1.05)	0.781
Urea (mg/dL)	1.00 (1,00; 1,01)	**0.017**	1.00 (1.00; 1.01)	**0.043**
Creatinine (mg/dL)	1.03 (0.93; 1.15)	0.486	1.05 (0.87; 1.28)	0.555
Urea to creatinine ratio	1.02 (1.00; 1.04)	**0.002**	1.01 (0.99; 1.03)	0.188
Neutrophil($x10^9$/L)	0.99 (0.93; 1.05)	0.781	1.00 (0.89; 1.13)	0.896
Lymphocyte($x10^9$/L)	0.99 (0.99; 1.00)	0.985	0.99 (0.99; 1.00)	0.260
Neutrophil lymphocyte ratio	1.00 (0.98; 1.01)	0.843	1.00 (0.98; 1.02)	0.559
C reactive protein(mg/dL)	1.00 (0.98; 1.03)	0.542	1.03 (0.99;1.07)	0.105
Lactate(mmol/L)	0.99 (0.97; 1.01)	0.473	0.99 (0.96; 1.01)	0.559
Diabetes	1.22 (0.66; 2.26)	0.510	1.58 (0.75; 3.34)	0.224
Hypertension	0.62 (0.62; 1.19)	0.155	0.60 (0.27; 1.33)	0.212
Chronic obstructive pulmonary disease	2.11 (0.88; 5.05)	0.091	1.40 (0.49; 3.99)	0.528
Chronic kidney disease	1.33 (0.53; 3.33)	0.534	1.70 (0.56; 5.20)	0.346
Cardiopathy	0.86 (0.42; 1.76)	0.686	0.76 (0.30; 1.90)	0.561
Dementia	2.40 (0.75; 7.68)	0.139	0.89 (0.22; 3.54)	0.874
Hypothyroidism	0.58 (0.21; 1.58)	0.288	0.65 (0.16; 2.50)	0.534
Cancer	1.17 (0.48; 2.83)	0.728	0.93 (0.34; 2.51)	0.887
No comorbidities	2.16 (0.82; 5.68)	0.115	1.93 (0.64; 5.77)	0.237
Invasive Mechanical Ventilation	0.81 (0.45; 1.46)	0.487	1.00 (0.47; 2.12)	0.992
Vasoactive drugs	0.91 (0.47; 1.75)	0.789	1.16 (0.49; 2.75)	0.727
Sedation	0.87 (0.47; 1.62)	0.676	1.02 (0.45; 2.30)	0.945
Hemodialysis	1.09 (0.40; 2.95)	0.865	1.75 (0.51; 5.99)	0.371
Edema (n, %)[2]	0.44 (0.19; 1.04)	0.062	1.22 (0.41; 3.61)	0.717
SOFA score	0.96 (0.89; 1.05)	0.440	0.96 (0.83; 1.11)	0.600
NUTRIC	1.05 (0.89; 1.24)	0.501	1.44 (0.97; 2.13)	0.065
NRS (continuous)	1.46 (1.15; 1.86)	**0.002***	1.02 (0.77; 1.35)	0.869
NRS (≥3 vs<3)	0.48 (0.07; 2.93)	0.427	0.36 (0.02; 4.88)	0.446
SGA (well-nourished vs undernourished)	6.00 (2.22; 11.17)	**<0.001***	2.56 (1.24; 5.27)	**0.011***

Hospital Length of Stay (days)	0.96 (0.92; 0.92)	0.999	0.97 (0.93; 1.01)	0.158

Model 1: Crude model; Model 2: adjusted model by sex, age and APACHE II. APACHE: Acute Physiology and Chronic Health Evaluation, NUTRIC: Nutrition Risk in Critically III, NRS: Nutritional Risk Screening, SGA: Subjective Global Assessment. *Significant p value (<0.05).

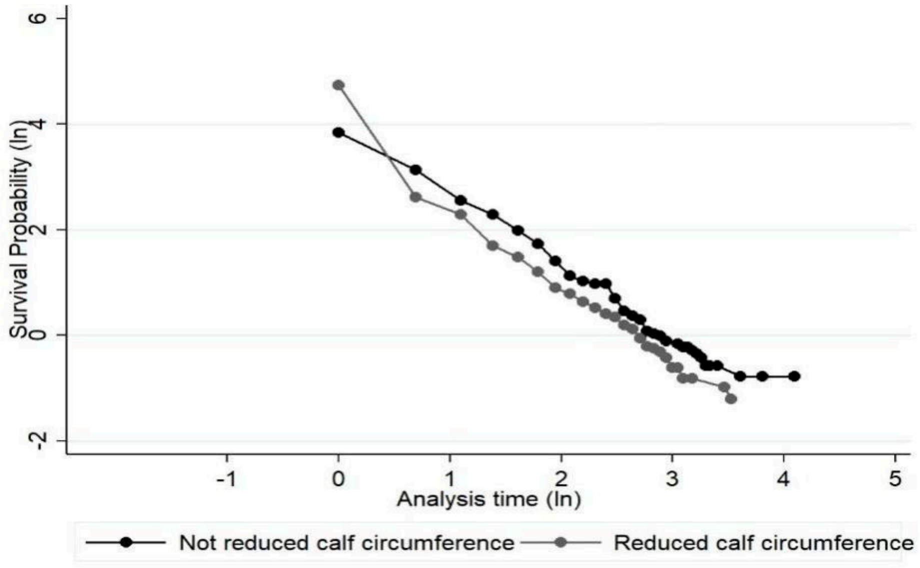

Figure 2. Survival curve of patients admitted at ICU with COVID-19 by calf circumference classification. Crude cox model.

Table 4. Survival model stratified by CCin patients admitted at ICU with COVID-19.

	**Model 1**		**Model 2**	
**Variables**	**OR (95% CI)**	**p**	**OR (95% CI)**	**p**
CC (reducedvs normal)	0.99 (0.55; 1.78)	0.991	1.11 (0.52; 2.37)	0.777

Crude cox model. Adjusted model by sex, age and APACHE II. *Significant p value (<0.05).
